# Melatonin supplementation reduces delirium incidence in critically ill patients: a systematic review and meta-analysis

**DOI:** 10.3389/fphar.2026.1728873

**Published:** 2026-01-12

**Authors:** Xiaoyu Wu, Panxin Hu, Haopeng Wu, Kai Zhang, Jianzhi Ying

**Affiliations:** 1 Department of Emergency Medicine, The First People’s Hospital of Taizhou, Taizhou, China; 2 Department of Critical Care Medicine, Second Affiliated Hospital, Zhejiang University School of Medicine, Hangzhou, China

**Keywords:** melatonin, delirium, critically ill, intensive care unit, meta-analysis

## Abstract

**Background:**

Delirium represents a common and severe neuropsychiatric complication among critically ill individuals, often leading to extended hospital stays, elevated mortality risk, and persistent cognitive decline. Disruption of circadian sleep-wake rhythms is a major pathogenic factor, implying that melatonin may hold therapeutic potential in this context. Nonetheless, evidence regarding the effectiveness of melatonin in preventing delirium among patients in intensive care unit (ICU) remains inconsistent.

**Objective:**

This meta-analysis aimed to systematically evaluate the effects of melatonin and melatonin receptor agonists on the incidence and duration of delirium, length of ICU stay, and mortality among adult critically ill patients.

**Methods:**

We conducted a systematic review and meta-analysis of randomized controlled trials (RCTs). A comprehensive search was performed in PubMed, Embase, Scopus, and Cochrane CENTRAL from inception until 10 October 2025. Only RCTs comparing melatonin or ramelteon with placebo or standard care in adult ICU patients were included. The primary outcome was the incidence of delirium. Secondary outcomes included duration of delirium, length of ICU stay, and overall mortality.

**Results:**

A total of 22 RCTs involving 3706 patients were included. Pooled analysis demonstrated that melatonin administration was associated with a significant reduction in the incidence of delirium compared to the control group (Risk Ratio [RR] 0.75, 95% Confidence Interval [CI] 0.63 to 0.90, P = 0.001, I^2^ = 50%). However, melatonin did not significantly affect the duration of delirium (Mean Difference [MD] 0.18 days, 95% CI -0.16 to 0.52, P = 0.56, I^2^ = 45%), length of ICU stay (MD -0.45 days, 95% CI -1.10 to 0.20, P = 0.09, I^2^ = 68%), or overall mortality (RR 0.92, 95% CI 0.79 to 1.06, P = 0.25, I^2^ = 0%).

**Conclusion:**

Among critically ill adults, melatonin supplementation is effective in reducing the incidence of delirium. However, it does not appear to shorten the duration of delirium, reduce ICU stay, or improve survival. These findings support the use of melatonin for delirium prevention but suggest its benefits may be limited to reducing occurrence rather than altering the course of established delirium or other clinical outcomes.

**Systematic Review Registration:**

https://osf.io/95je7.

## Background

Among critically ill patients, delirium constitutes a major neuropsychiatric disorder marked by abrupt alterations in attention, consciousness, and cognitive function (Wilcox et al., 2021). The reported prevalence in intensive care units (ICUs) ranges from 20% to 80%, depending on patient populations, illness severity, and detection methods (Stollings et al., 2021). Development of delirium in critically ill patients is linked to numerous unfavorable outcomes, such as higher short- and long-term mortality, longer durations of mechanical ventilation, and extended ICU or hospital admission (Salluh et al., 2015; Nassar et al., 2023). Furthermore, delirium is predictive of long-term cognitive impairment resembling dementia and reduced quality of life after discharge, which imposes a significant burden on patients, families, and healthcare systems (Goldberg et al., 2020).

Current prevention and management strategies for ICU delirium emphasize non-pharmacological interventions, such as early mobilization, optimization of sleep-wake cycles, minimization of sedation, and environmental adjustments (Devlin et al., 2018). Pharmacological prophylaxis and treatment remain controversial, with no agent showing definitive benefit in large randomized controlled trials. Antipsychotics have been widely used, yet recent evidence does not support their routine use for either prevention or treatment (Nikooie et al., 2019).

Melatonin, a naturally occurring hormone that governs circadian cycles and sleep regulation, has been suggested as a potential preventive or therapeutic agent for ICU-associated delirium (Cipolla-Neto and Amaral, 2018). Several randomized controlled trials (RCTs) have investigated melatonin or melatonin receptor agonists for delirium prevention in different hospitalized populations, including the critically ill patients. Previous systematic reviews and meta-analyses have suggested that melatonin supplementation may be beneficial in reducing delirium incidence among critically ill patients. However, recent large-scale RCTs failed to demonstrate significant benefits of melatonin supplementation on delirium incidence in critically ill patients (Burry et al., 2025; Mekontso Dessap et al., 2025). These findings have introduced uncertainty regarding the true efficacy of melatonin for delirium prevention and have raised questions about the robustness of previous meta-analytic conclusions.

Given the burden of delirium in the ICU, the limited efficacy of current pharmacological strategies, and the growing interest in melatonin as a potentially safe preventive or therapeutic agent, a comprehensive and updated meta-analysis of randomized controlled trials is warranted. Such analysis could clarify the overall effect of melatonin on delirium incidence among critically ill patients, identify subgroups most likely to benefit, and inform future clinical guidelines and research directions.

## Methods

### Search strategy and study selection

This review and meta-analysis adhered to the PRISMA framework to ensure methodological transparency and reproducibility (Page et al., 2021), with the completed checklist provided in Supplementary Material 1. The research protocol was prospectively recorded on the Open Science Framework platform (https://osf.io/95je7). As this study involved analysis of previously published data, institutional review board approval was not required.

Independent investigators systematically searched PubMed, Embase, Scopus, and Cochrane CENTRAL databases from their inception to 10 October 2025. The search included keywords such as “intensive care unit”, “critically ill”, “melatonin”, “delirium”, and “randomized controlled trial”. A complete description of the search strategies is available in Supplementary Material 2.

Eligibility criteria encompassed:

1. Population: Adult ICU patients (≥18 years).
2. Intervention: Administration of exogenous melatonin or melatonin receptor agonists (e.g., ramelteon) at any dosage and duration.
3. Comparator: Placebo or standard care without melatonin supplementation.
4. Outcomes: Primary outcome was delirium incidence during ICU stay, defined by a positive assessment using validated tools such as Confusion Assessment Method for the ICU (CAM-ICU), Intensive Care Delirium Screening Checklist (ICDSC), or by clinician diagnosis. Secondary outcomes included duration of delirium, length of ICU stay, and overall mortality.
5. Study Design: RCTs published in English.

Exclusion criteria included: (1) non-randomized studies; (2) studies involving pediatric populations (<18 years); (3) studies where melatonin was administered for conditions other than delirium prevention; (4) studies with insufficient data for meta-analysis; and (5) duplicate publications or secondary analyses of already included trials.

**FIGURE 1:**
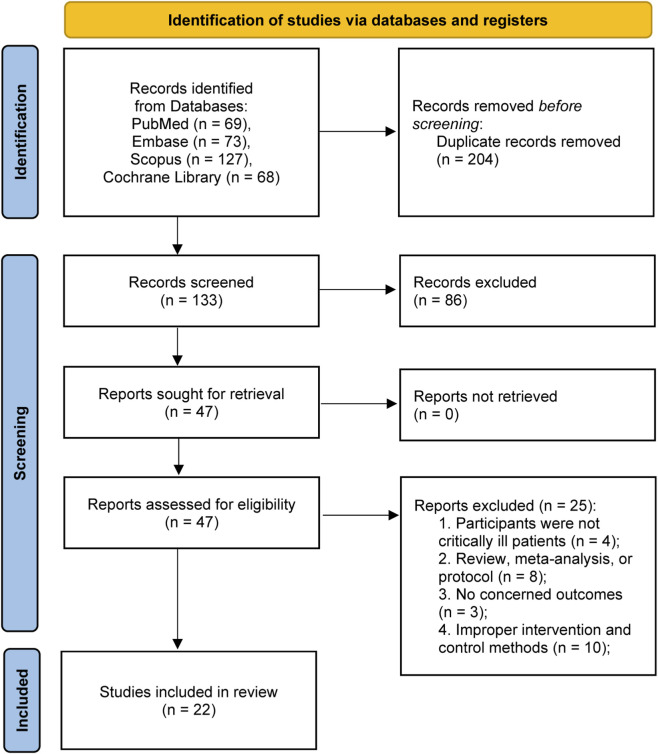
PRISMA 2020 flow diagram for this meta-analysis.

Initial screening of all identified citations occurred independently by two reviewers who evaluated titles and abstracts in accordance with established eligibility parameters. Potentially eligible citations proceeded to comprehensive full-text examination. The same two independent reviewers subsequently conducted detailed full-text analyses to establish final study inclusion status. Reviewer disagreements were settled through collegial discussion. A PRISMA flow diagram (Figure 1) was constructed to illustrate the study selection pathway, quantifying studies at each decision point and documenting specific exclusion rationales.

### Data extraction

Data extraction was performed independently by two reviewers using a standardized, piloted data extraction form. The extracted data included: study and participant characteristics, intervention details, comparison group details; and outcome data for all predefined outcomes. For continuous outcomes (duration of delirium, length of ICU stay), we extracted means and standard deviations. For dichotomous outcomes (incidence of delirium, overall mortality), we extracted event counts in each group. When data were reported in different formats or were missing, we attempted to contact the corresponding authors for clarification.

### Quality assessment

Parallel independent assessment of trial methodological quality was undertaken using the Cochrane Risk of Bias 2.0 tool (Sterne et al., 2019), which evaluates five domains: (1) bias inherent to the randomization procedure; (2) bias consequent to deviation from protocol; (3) bias attributable to incomplete outcome data; (4) bias in outcome ascertainment; and (5) bias arising from selective outcome reporting. Categorical risk assignments (“low risk”, “some concerns”, “high risk”) were applied to each domain.

Dissemination bias was evaluated through Egger’s parametric test and graphical examination of funnel plot symmetry (Egger et al., 1997). Where funnel asymmetry was indicative of small-study effects, trim-and-fill adjustment was performed to evaluate estimate durability under alternative bias assumptions (Duval and Tweedie, 2000). Conflicting assessments across all review phases were harmonized through iterative team consensus.

### Statistical synthesis and analysis

Meta-analytic computations were implemented using Review Manager version 5.4 (Cochrane Collaboration) and R statistical software (“meta” and “robvis” package). Dichotomous outcomes (incidence of delirium incidence, overall mortality) were synthesized as pooled risk ratios (RRs) with 95% confidence intervals (CIs) using the Mantel-Haenszel method. Continuous outcomes (delirium duration, ICU length of stay) were synthesized as pooled mean differences (MDs) with 95% CIs using the Inverse-Variance method.

Interstudy heterogeneity was evaluated via the I^2^ statistic and Cochran’s Q test (Higgins et al., 2003). Heterogeneity magnitude interpretation employed established benchmarks: I^2^ values of 25%, 50%, and 75% denoting low, moderate, and high heterogeneity, respectively. Analytical model selection was contingent upon heterogeneity: fixed-effects models when I^2^ < 50%, and random-effects models (DerSimonian-Laird method) when I^2^ ≥ 50%. Prospectively defined subgroup stratification examined primary outcome heterogeneity sources across three dimensions: patient type (surgical vs. non-surgical), age (elderly vs. non-elderly, elderly patient was defined as ≥60 years old), and melatonin dose [<3 mg vs. ≥ 3 mg daily, according to the DEMEL trial (Mekontso Dessap et al., 2025)]. Sensitivity analysis via iterative single-study omission was undertaken to evaluate finding robustness. Two-tailed statistical significance was defined as P < 0.05 (P < 0.10 for heterogeneity testing).

## Results

### Study selection and study characteristics

Comprehensive electronic database interrogation identified 337 initial bibliographic references. After deduplication (204 records excluded), 133 distinct citations underwent preliminary screening based on title and abstract content. Full-text evaluation was subsequently performed on 47 potentially eligible publications. The final analytic cohort comprised 22 randomized controlled trials involving 3,706 critically ill adult patients, all of which fulfilled the predetermined eligibility specifications (Burry et al., 2025; Mekontso Dessap et al., 2025; Abbasi et al., 2018; Akhileshwar et al., 2025; Bandyopadhyay et al., 2024; Bourne et al., 2008; Dianatkhah et al., 2015; Duggappa et al., 2016; Ford et al., 2019; Foreman et al., 2015; Gandolfi et al., 2020; Gupta et al., 2022; Hatta et al., 2014; Jaiswal et al., 2019; Javaherforoosh Zadeh et al., 2021; Javaherforooshzadeh et al., 2023; Mahrose et al., 2021; Naderi-Behdani et al., 2022; Nishikimi et al., 2018; Shi, 2021; Wibrow et al., 2022; Yin et al., 2022). The complete study selection workflow, including detailed justifications for full-text stage attrition, is depicted in an accompanying PRISMA-formatted selection diagram (Figure 1).

The 22 included RCTs were published between 2008 and 2025. The sample sizes of the individual studies ranged from 12 to 841 participants. All studies enrolled adult patients admitted to medical (Bourne et al., 2008; Duggappa et al., 2016; Hatta et al., 2014; Nishikimi et al., 2018), surgical (Dianatkhah et al., 2015; Ford et al., 2019; Gupta et al., 2022; Jaiswal et al., 2019; Javaherforoosh Zadeh et al., 2021; Javaherforooshzadeh et al., 2023; Mahrose et al., 2021; Shi, 2021), or mixed ICUs (Burry et al., 2025; Mekontso Dessap et al., 2025; Abbasi et al., 2018; Akhileshwar et al., 2025; Bandyopadhyay et al., 2024; Foreman et al., 2015; Gandolfi et al., 2020; Naderi-Behdani et al., 2022; Wibrow et al., 2022; Yin et al., 2022). The intervention consisted of exogenous melatonin administered orally or via enteral tube, with doses ranging from 0.3 mg to 10 mg, primarily given at nighttime. The control groups received either a matching placebo or standard care without melatonin. The most common tool for delirium assessment was the CAM-ICU. Key characteristics of the included studies, including patient demographics, intervention details, and outcome measures, are summarized in Table 1.

**TABLE 1:** Characteristics of included studies.

Study	N	Characteristics	Intervention	Control	Definition of delirium
Bourne 2008	12/12	Adult ICU patients with acute respiratory failure requiring mechanical ventilation	Melatonin (10 mg) for 4 days	Placebo	NR
Hatta 2014	33/34	Elderly patients (≥65 years) admitted into ICUs	Melatonin (8 mg) for 7 days	Placebo	DSM-IV criteria
Dianatkhah 2015	66/71	Elective CABG adult patients in ICU	Melatonin (3 mg), started 3 days before surgery, until discharge	Oxazepam (10 mg)	Nursingrecords
Foreman 2015	6/6	Adult patients admitted into NICU	Melatonin (3 mg) for 7 days	Standard care	Nursingrecords
Vijayakumar 2016	26/30	Adult patients with organophosphorus compound poisoning admitted into ICU	Melatonin (3 mg) during ICU stay	Placebo	CAM-ICU
Abbasi 2018	67/70	Adult critically ill patients in ICU	Melatonin (3 mg) for 5 days	Placebo	CAM-ICU
Nishikimi 2018	45/43	Adult critically ill patients in ICU	Ramelteon (8 mg) during ICU stay	Placebo	CAM-ICU
Jaiswal 2019	59/58	Adult patients admitted into ICU after pulmonary thromboendarterectomy	Ramelteon (8 mg) for 6 days	Placebo	CAM-ICU
Ford 2019	105/105	Adult patients (≥50 years) admitted into ICU after elective cardiac surgery	Melatonin (3 mg) for 7 days	Placebo	CAM-ICU
Gandolfi 2020	102/101	Adult critically ill patients in ICU	Melatonin (10 mg) for 7 days	Placebo	ICDSC
Shi 2021	148/149	Patients (≥60 years) admitted into ICU after PCI	Melatonin (3 mg) for 7 days	Placebo	CAM-ICU
Mahrose 2021	55/55	Patients (≥60 years) admitted into ICU after CABG	Melatonin (5 mg) for 4 days	No placebo	CAM-ICU
Zadeh 2021	30/30	Adult patients in cardiovascular ICU after CABG	Melatonin (3 mg) for 3 days	Placebo	CAM-ICU
Pro-MEDIC trial 2022	419/422	Adult critically ill patients in ICU	Melatonin (2 mg) for 14 days	Placebo	CAM-ICU
Gupta 2022	70/70	Adult patients admitted into ICU after operation	Melatonin (5 mg) until ICU discharge	Placebo	CAM-ICU
Naderi-behdani 2022	48/48	Adult critically ill patients in ICU	Melatonin (6 mg) for 3 days	Placebo	CAM-ICU
Yin 2022	248/249	Patients (≥60 years) with acute heart failure in ICU	Melatonin (2 mg) for 7 days	Placebo	CAM-ICU
Javaherforooshzadeh 2023	40/40	Adult patients in ICU after CABG	Melatonin (3 mg) for 5 days	No placebo	CAM-ICU
Bandyopadhyay 2024	54/54	Adult critically ill patients in ICU	Melatonin (3 mg) for 7 days	Placebo	CAM-ICU
Burry 2025	46/24	Adult critically ill patients in ICU	Melatonin (0.5 or 2 mg) for 14 days	Placebo	CAM-ICU
Akhileshwar 2025	27/28	Adult critically ill patients in ICU	Melatonin (8 mg) until ICU discharge	Placebo	CAM-ICU
DEMEL trial 2025	147/154	Adult critically ill patients receiving invasive mechanical ventilation in ICU	Melatonin (0.3 or 3 mg) for 14 days	Placebo	CAM-ICU

N, number of participants (experimental/control); ICU, intensive care unit; CABG, coronary artery bypass grafting; CAM-ICU, confusion assessment method for the intensive care unit; ICDSC, intensive care delirium screening checklist; DSM-IV, diagnostic and statistical manual of mental disorders, fourth edition.

### Quality assessment

**FIGURE 2:**
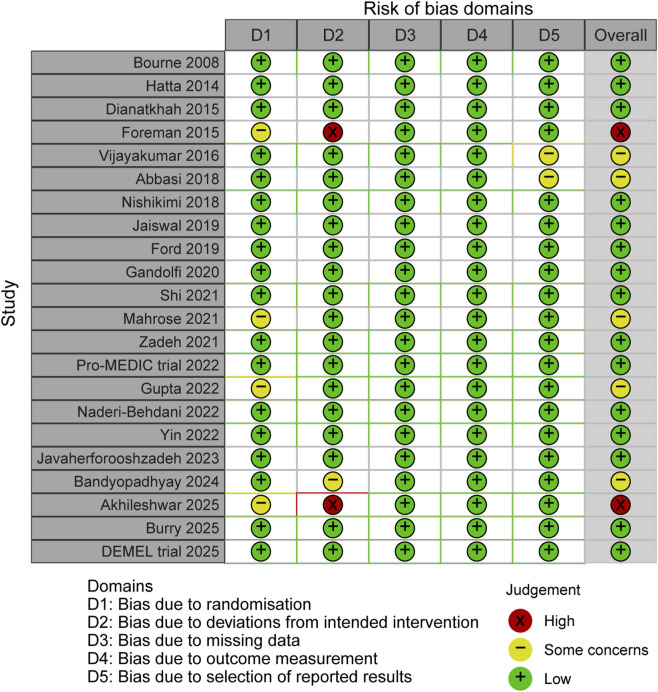
Assessment of quality by the Cochrane risk of bias tool.

Quality assessment findings employing the Cochrane Risk of Bias framework are graphically represented in Figure 2. The majority of the 22 enrolled trials demonstrated low-to-moderate risk classifications across most evaluation domains. Notably, 15 trials received “low risk” ratings across all five domains, reflecting robust methodological execution. Five trials exhibited “some concerns” in at least one domain, predominantly attributable to inadequate randomization methodology or selective outcome reporting. Only two trials were classified as “high risk”, chiefly reflecting protocol deviations during intervention delivery. Collectively, trial methodological quality was deemed satisfactory, furnishing dependable information for meta-analytic synthesis.

Dissemination bias evaluation employed funnel plot visualization and Egger regression analysis (Supplementary Material 3, Supplementary Figure S1). Asymmetrical funnel patterns indicative of potential publication bias emerged for delirium incidence and ICU length-of-stay outcomes. Trim-and-fill adjustment methodology was employed to assess estimate robustness under alternative bias scenarios. Following theoretical imputation of suppressed studies, adjusted pooled estimates demonstrated consistency with the original unadjusted estimates (Supplementary Material 3, Supplementary Figures S2, S3), indicating that dissemination bias did not substantially alter our meta-analytic inferences.

### Primary outcome

**FIGURE 3:**
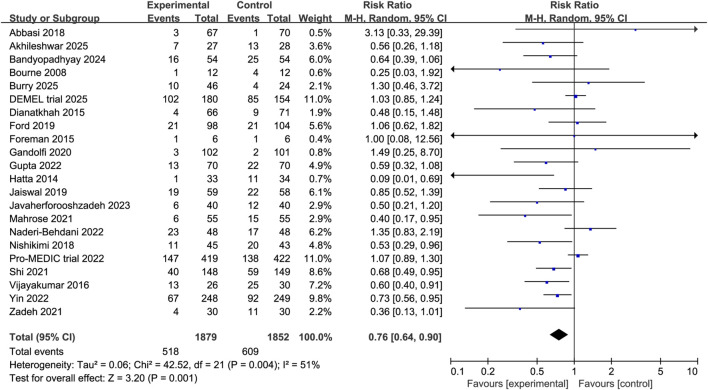
Forest plot showing the association between melatonin and incidence of delirium.

Pooled data from all 22 RCTs demonstrated that melatonin was associated with a statistically significant reduction in the incidence of delirium compared to the control group (RR 0.75, 95% CI 0.63 to 0.90, P = 0.001, I^2^ = 50%, Figure 3). Sensitivity analysis performed by sequentially excluding each study confirmed the stability of the main result.

**FIGURE 4:**
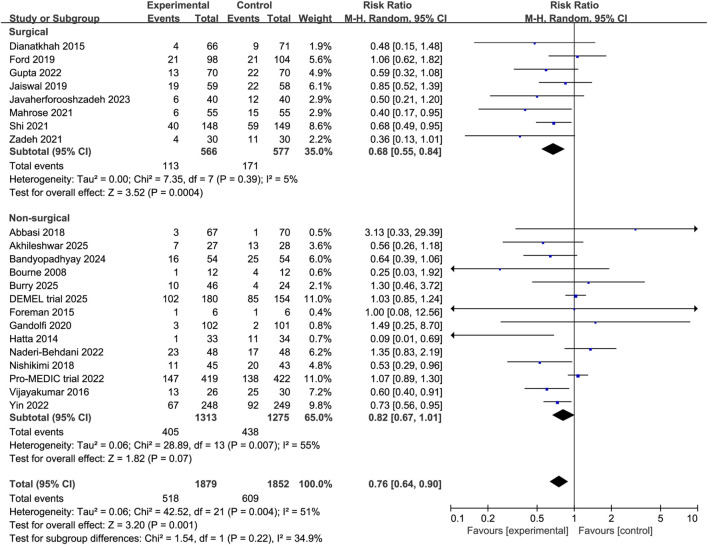
Forest plot showing the subgroup analysis stratified by patient type (surgical vs. non-surgical).

**FIGURE 5:**
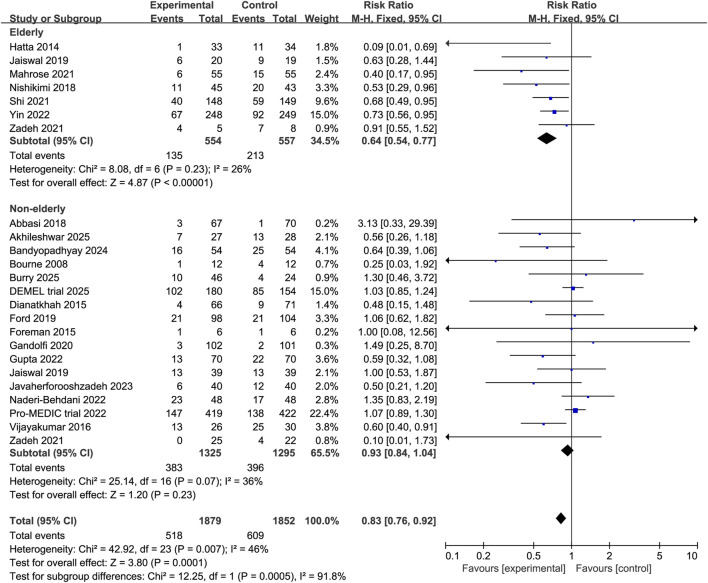
Forest plot showing the subgroup analysis stratified by age (elderly vs. non-elderly).

**FIGURE 6:**
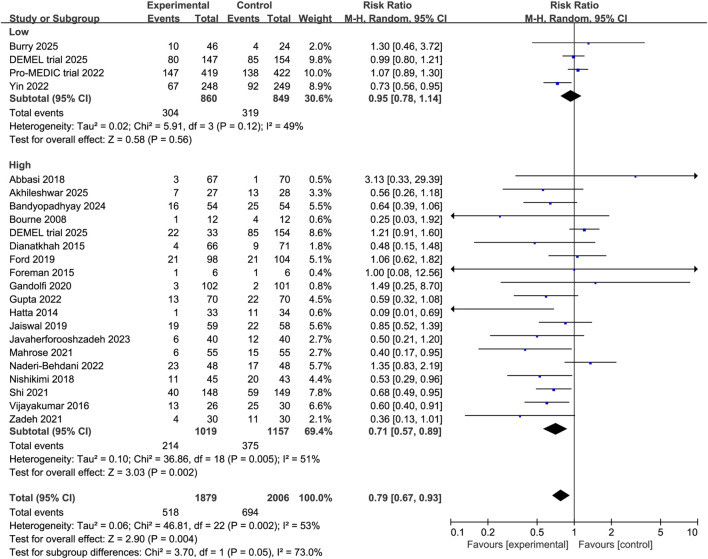
Forest plot showing the subgroup analysis stratified by melatonin dose (<3 mg vs. ≥ 3 mg daily).

Subgroup analyses revealed that melatonin significantly reduced the incidence of delirium among surgical patients (RR 0.68, 95% CI 0.55 to 0.84, P = 0.0004, I^2^ = 5%, Figure 4) and elderly patients (RR 0.64, 95% CI 0.54 to 0.77, P < 0.00001, I^2^ = 26%, Figure 5). In contrast, no significant effect was observed in non-surgical or non-elderly subgroups (non-surgical: RR 0.82, 95% CI 0.67 to 1.01, P = 0.07, I^2^ = 55%, Figure 4; non-elderly: RR 0.93, 95% CI 0.84 to 1.04, P = 0.23, I^2^ = 36%; Figure 5). Moreover, analyses stratified by dosage indicated that higher doses of melatonin were associated with a significant reduction in delirium incidence (RR 0.71, 95% CI 0.57 to 0.89, P = 0.002, I^2^ = 51%, Figure 6), whereas lower doses showed no statistically significant benefit (RR 0.95, 95% CI 0.78 to 1.14, P = 0.56, I^2^ = 49%, Figure 6). These findings suggest a potential population-specific effectiveness and dose-response relationship of melatonin in delirium prevention. Moreover, no change in the direction of results in the sensitivity analysis that omit every single study at a time, indicating the good robustness (Supplementary Material 3, Supplementary Figure S4).

### Secondary outcomes

**FIGURE 7:**
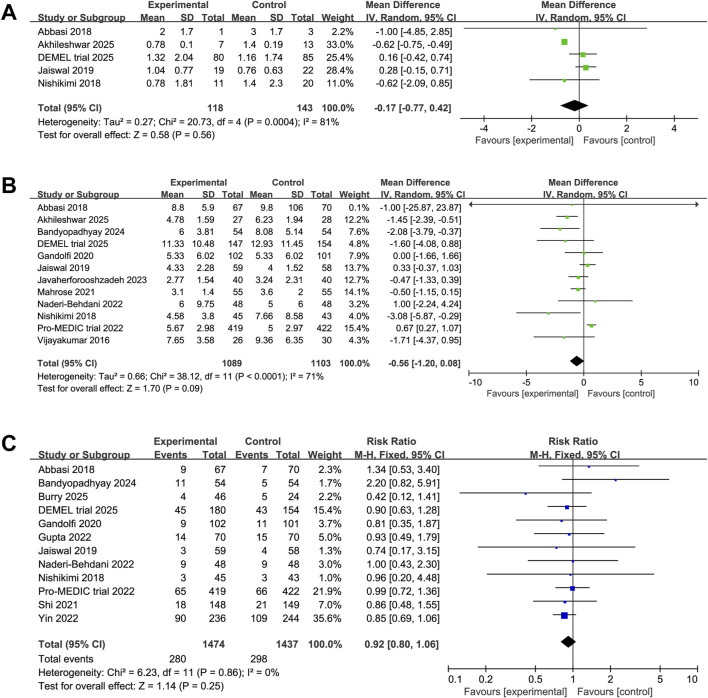
Forest plot showing the association between melatonin and **(A)** duration of delirium, **(B)** length of ICU stay, **(C)** mortality.

Pooled analysis of five studies revealed no significant difference for duration of delirium between the melatonin and control groups (MD 0.18 days, 95% CI -0.16 to 0.52, P = 0.56, I^2^ = 45%, Figure 7A). Regarding length of ICU stay, pooled analysis of 12 studies showed a trend toward reduction in the melatonin group compared with controls (MD -0.45 days, 95% CI -1.10 to 0.20, P = 0.09, I^2^ = 68%, Figure 7B), although this did not reach statistical significance. Moreover, no significant difference was found in overall mortality between the melatonin and control groups (RR 0.92, 95% CI 0.79 to 1.06, P = 0.25, I^2^ = 0%, Figure 7C).

Sensitivity analysis was conducted to assess the robustness of the findings by sequentially excluding each study and recalculating the pooled estimates. Removal of Pro-MEDIC trial (Wibrow et al., 2022) from the analysis resulted in a statistically significant reduction in ICU length of stay with melatonin use (MD -0.73 days, 95% CI -1.34 to −0.13, P = 0.02, I^2^ = 46%, Supplementary Material 3, Supplementary Figure S5), whereas findings for delirium duration and mortality remained unchanged (Supplementary Material 3, Supplementary Figures S6, S7).

## Discussion

### Summary of main findings

This meta-analysis including 22 RCTs, demonstrated that melatonin administration significantly reduced the incidence of delirium among critically ill patients. However, melatonin did not shorten the duration of delirium, reduce ICU length of stay, or improve mortality. Subgroup analyses revealed that melatonin was particularly effective in postoperative and elderly populations, while no significant benefit was observed among non-surgical or younger patients. Moreover, higher doses of melatonin appeared to be necessary to achieve a preventive effect, whereas lower doses showed no significant impact.

### Clinical implications and mechanistic considerations

The findings indicated that melatonin reduces the incidence of delirium but not the duration of delirium, suggesting its primary benefit may lie in prevention rather than treatment. Melatonin exerts its protective effects against delirium through multiple biological mechanisms. As a potent endogenous antioxidant, melatonin scavenges free radicals and enhances antioxidant enzyme activity, thereby attenuating oxidative stress—a key pathophysiological mechanism implicated in delirium development (Kołodziejska et al., 2025). Furthermore, melatonin’s immunomodulatory properties may suppress excessive inflammatory responses, including proinflammatory cytokine production, which is strongly associated with delirium pathogenesis in the ICU setting (Hume et al., 2024). Additionally, melatonin regulates circadian rhythm homeostasis and promotes sleep architecture, both of which are frequently disrupted in critically ill patients and contribute to delirium risk. Critically ill patients frequently experience circadian disruption due to continuous light exposure, noise, sedative and opioid use, and sleep fragmentation (Boyko et al., 2012; Kamdar et al., 2015). By restoring sleep–wake cycles and modulating inflammatory and mitochondrial pathways, melatonin may enhance neurocognitive stability and thereby reduce the likelihood of delirium onset (Andersen et al., 2016). However, once delirium develops, downstream neuroinflammatory, neurotransmitter, and network dysconnectivity processes may have progressed beyond the preventive capacity of melatonin, which could explain its limited effect on delirium duration or on global clinical outcomes (Wada et al., 2023).

The subgroup findings highlight that patient characteristics and clinical context may influence melatonin’s efficacy. The significant benefit observed among postoperative and elderly patients aligns with the notion that these groups are more susceptible to sleep disruption, circadian misalignment, and neuroinflammation. Surgical stress, anesthesia, and postoperative pain can further aggravate endogenous melatonin suppression and circadian phase shifts, making exogenous supplementation particularly plausible in these populations (Ortiz, 2020; Gao et al., 2021). In contrast, non-surgical and younger patients may experience delirium through multifactorial mechanisms less directly related to circadian rhythm disturbance, which could explain the lack of observed benefit in these subgroups.

Furthermore, our subgroup analysis further suggests that melatonin’s effect is dose-dependent, with higher doses being required to achieve neuroprotective and circadian-stabilizing effects. This is consistent with pharmacologic considerations that critical illness can alter gastrointestinal absorption, hepatic metabolism, and blood–brain barrier permeability, potentially necessitating higher or scheduled evening dosing to achieve target exposure (Zisapel, 2018). Standardizing dosage regimens, formulation, and administration timing in future trials may help clarify the optimal melatonin strategy for delirium prevention in the ICU settings.

Our finding that higher melatonin doses (≥3 mg daily) were associated with greater preventive benefit appears to differ from the conclusion of Yang et al. (2020), whose network meta-analysis suggested that very low-dose melatonin (0.5 mg) may be more effective than higher doses such as 5 mg. However, this apparent discrepancy should be interpreted with caution. The analysis by Yang et al. included only six RCTs and, importantly, only one trial that evaluated a low-dose regimen; thus, the comparative effect estimates were derived from an evidence network with extremely sparse and imbalanced dose data. As Rodrigues et al. (2020) noted in their methodological critique, the limited number of studies and the lack of population-specific analyses, particularly among older adults, who demonstrate different circadian physiology and delirium susceptibility.

These considerations align with our present subgroup analyses, which stratified patients by age and demonstrated that elderly ICU patients benefited most clearly from melatonin supplementation. This finding supports the argument raised by Rodrigues et al. (2020) that age-specific analyses are essential for accurately characterizing melatonin’s effects. Taken together, the limited low-dose evidence in Yang et al. (2020) and the subsequent methodological insights by Rodrigues et al. (2020) suggest that the higher-dose benefit observed in our study is biologically plausible and likely reflects a more robust and representative evidence base among critically ill adults.

Although melatonin showed promise in reducing delirium incidence, it did not translate into reductions in length of ICU stay or mortality. This may be attributed to the multifactorial nature of ICU outcomes, which are influenced by numerous factors beyond delirium alone, such as disease severity, comorbidities, and treatment complexity (Hermann et al., 2021). Moreover, the included studies varied in melatonin dosage, duration, and timing of administration, which may have introduced heterogeneity and attenuated the observable clinical effects.

### Comparison to the previous literature

Our meta-analytic findings demonstrate consistency with the extant literature, substantiating melatonin as an effective delirium-preventive modality in critically ill populations. A contemporaneous meta-analysis by Tang et al. examined whether exogenous melatonin administration in ICU environments enhances patient-centered clinical endpoints, concluding that melatonin may attenuate delirium risk (based on 16 RCTs) yet exerts negligible influence on delirium duration (5 RCTs) (Tang et al., 2025). However, Lakbar et al. analyzed 6 RCTs with low risk of bias, indicated that melatonin does not reduce delirium incidence in critically ill patients (Lakbar et al., 2025). Contrastingly, Zhao et al. documented differential efficacy: significant delirium incidence reduction in specialized ICU cohorts, but no meaningful benefit in general ICU populations (Zhao et al., 2024). By incorporating the latest RCTs, our meta-analysis provides further confirmation that melatonin administration is associated with a reduced incidence of delirium. We also identified specific patient populations benefiting from melatonin: surgical and elderly patients. Furthermore, our analysis reveals a dose-dependent relationship, with only higher-dose regimens achieving significance. The two most recent large-scale RCTs (Burry et al., 2025; Mekontso Dessap et al., 2025) reported no significant effect of melatonin on delirium. However, these trials primarily used low-dose melatonin (0.3–2 mg daily). Given increasing evidence that critically ill patients may require higher doses to overcome circadian disruption and altered pharmacokinetics, the use of low-dose regimens may account for their neutral findings. Our subgroup analysis supports this interpretation, demonstrating that higher doses (≥3 mg daily) were associated with a significant reduction in delirium incidence, whereas lower doses were not. These dose differences likely contribute to the divergent conclusions observed across studies.

### Strength and limitations

This meta-analysis has several strengths. It provides the most comprehensive synthesis to date of RCTs evaluating melatonin for delirium prevention in critically ill patients and includes subgroup analyses that clarify population- and dose-specific effects. However, some limitations should be acknowledged. First of all, most studies had relatively small sample sizes and short follow-up durations. The lack of long-term follow-up data in most studies precludes assessment of melatonin’s effects on long-term cognitive outcomes, a matter of increasing clinical importance given the associated morbidity of ICU-acquired cognitive impairment (Voiriot et al., 2022). Secondly, considerable heterogeneity was observed across the included studies, likely reflecting differences in melatonin dosing protocols, variability in administration timing and duration, differences in delirium assessment tools and diagnostic criteria, heterogeneous ICU populations, and variable baseline delirium risk across studies. Furthermore, clinical endpoints such as length of ICU stay and overall mortality were often secondary outcomes with limited power, constraining inferences about downstream effects.

### Future research directions

Future research should prioritize large, multicenter trials that standardize delirium assessment (e.g., CAM-ICU with defined frequency), incorporate objective sleep and circadian measures (actigraphy or validated sleep scales), and test dosing and formulation strategies. Trials should prespecify clinically meaningful outcomes beyond incident delirium, such as delirium duration, rescue medication use, sedation exposure, ventilator-free days, and post-discharge cognitive function (Voiriot et al., 2022). Stratified analyses in high-risk subgroups (elderly patients, sepsis, mechanically ventilated patients, patients receiving benzodiazepines) may identify populations most likely to benefit. Dose-optimization trials systematically evaluating melatonin plasma concentrations in relation to clinical outcomes are needed to establish evidence-based dosing recommendations. Moreover, investigation of melatonin’s efficacy when administered as part of multimodal delirium prevention bundles, compared to melatonin monotherapy, would clarify its complementary role within comprehensive prevention strategies.

## Conclusion

In summary, melatonin appears to be an effective and safe preventive intervention for delirium in critically ill patients, particularly in postoperative and elderly populations and when administered at higher doses. Nevertheless, melatonin does not seem to reduce delirium duration, length of ICU stay, or mortality. Further large-scale, high-quality RCTs are warranted to confirm these findings and to determine the optimal dosage, timing, and duration of melatonin therapy for delirium prevention in the ICU.

## Data Availability

The original contributions presented in the study are included in the article/Supplementary Material, further inquiries can be directed to the corresponding author.
