# Supplementary material for: Melatonin supplementation reduces delirium incidence in critically ill patients: a systematic review and meta-analysis
Source: Front Pharmacol. 2026 Jan 12;17:1728873. doi: 10.3389/fphar.2026.1728873 (PMC12832284; doi:10.3389/fphar.2026.1728873)
Supplement: Supplementary file 3 [file Supplementaryfile3.pdf]

Supplementary Material 3: Publication bias assessment by funnel plot and Egger's test, forest plot after trim-and-fill adjustment, and sensitivity analysis.

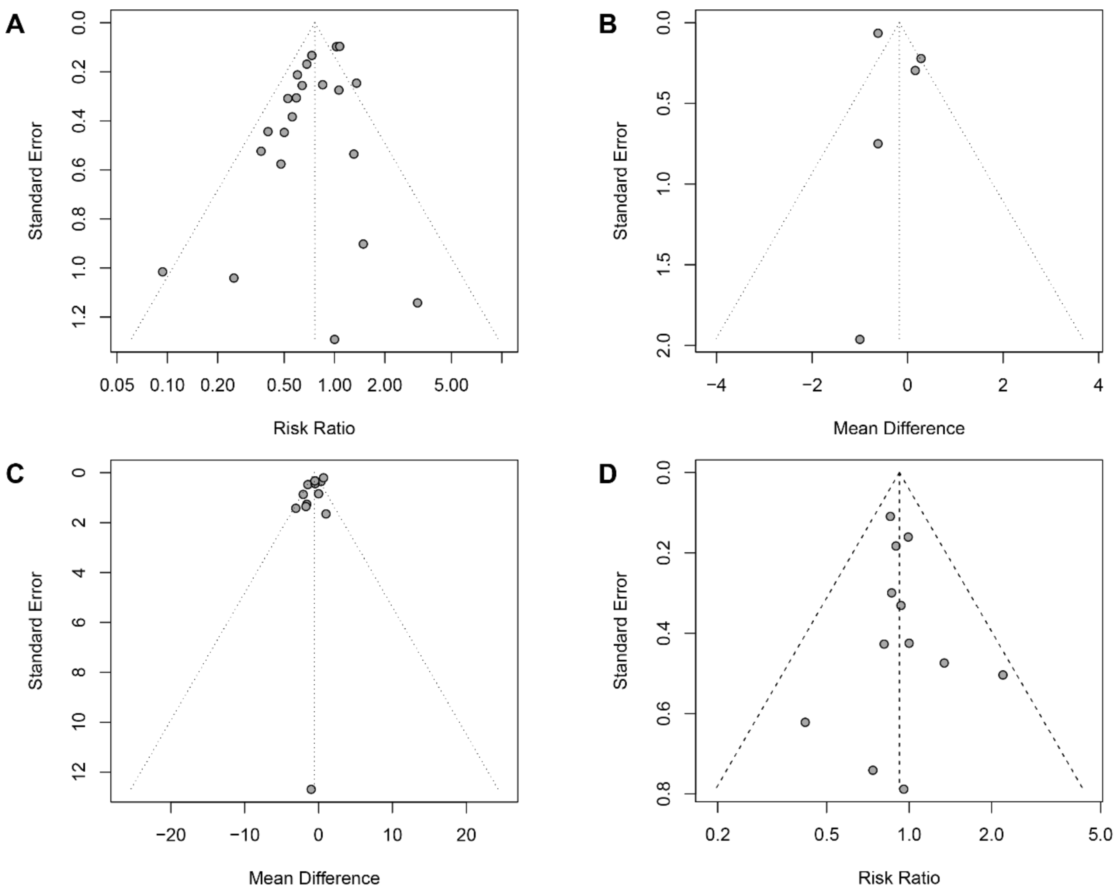

Figure S1: Funnel plot for (A) delirium, Egger's test P=0.0234, (B) duration of delirium, Egger's test P=0.3517, (C) length of ICU stay, Egger's test P=0.0441, (D) overall mortality, Egger's test P=0.5986

Study	logRR	SE	Exp Total	Con Total	Weight	Risk Ratio IV, Random, 95% CI
Abbasi 2018	1.1424	1.1420	67	70	0.5%	3.13 [0.33; 29.39]
Akhileshwar 2025	−0.5827	0.3834	27	28	3.4%	0.56 [0.26; 1.18]
Bandyopadhyay 2024	−0.4463	0.2559	54	54	5.6%	0.64 [0.39; 1.06]
Bourne 2008	−1.3863	1.0408	12	12	0.6%	0.25 [0.03; 1.92]
Burry 2025	0.2657	0.5353	46	24	2.0%	1.30 [0.46; 3.72]
DEMEL trial 2025	0.0263	0.0976	180	154	9.8%	1.03 [0.85; 1.24]
Dianatkhah 2015	−0.7379	0.5761	66	71	1.8%	0.48 [0.15; 1.48]
Ford 2019	0.0594	0.2746	98	104	5.2%	1.06 [0.62; 1.82]
Foreman 2015	0.0000	1.2910	6	6	0.4%	1.00 [0.08; 12.56]
Gandolfi 2020	0.3956	0.9020	102	101	0.8%	1.49 [0.25; 8.70]
Gupta 2022	−0.5261	0.3063	70	70	4.6%	0.59 [0.32; 1.08]
Hatta 2014	−2.3680	1.0155	33	34	0.7%	0.09 [0.01; 0.69]
Jaiswal 2019	−0.1637	0.2528	59	58	5.6%	0.85 [0.52; 1.39]
Javaherforooshzadeh 2023	−0.6931	0.4472	40	40	2.7%	0.50 [0.21; 1.20]
Mahrose 2021	−0.9163	0.4438	55	55	2.8%	0.40 [0.17; 0.95]
Naderi−Behdani 2022	0.3023	0.2462	48	48	5.8%	1.35 [0.83; 2.19]
Nishikimi 2018	−0.6433	0.3089	45	43	4.5%	0.53 [0.29; 0.96]
Pro−MEDIC trial 2022	0.0703	0.0964	419	422	9.9%	1.07 [0.89; 1.30]
Shi 2021	−0.3819	0.1688	148	149	7.8%	0.68 [0.49; 0.95]
Vijayakumar 2016	−0.5108	0.2124	26	30	6.6%	0.60 [0.40; 0.91]
Yin 2022	−0.3131	0.1332	248	249	8.8%	0.73 [0.56; 0.95]
Zadeh 2021	−1.0116	0.5237	30	30	2.1%	0.36 [0.13; 1.01]
Filled: Dianatkhah 2015	0.4884	0.5761	66	71	1.8%	1.63 [0.53; 5.04]
Filled: Mahrose 2021	0.6668	0.4438	55	55	2.8%	1.95 [0.82; 4.65]
Filled: Zadeh 2021	0.7621	0.5237	30	30	2.1%	2.14 [0.77; 5.98]
Filled: Bourne 2008	1.1368	1.0408	12	12	0.6%	3.12 [0.41; 23.97]
Filled: Hatta 2014	2.1186	1.0155	33	34	0.7%	8.32 [1.14; 60.88]
**Total (95% CI)**			**2075**	**2054**	**100.0%**	**0.83 [0.70; 0.97]**

Heterogeneity: $Tau^2 = 0.0634$; $Chi^2 = 55.92$, df = 26 ($P < 0.01$); $I^2 = 54\%$

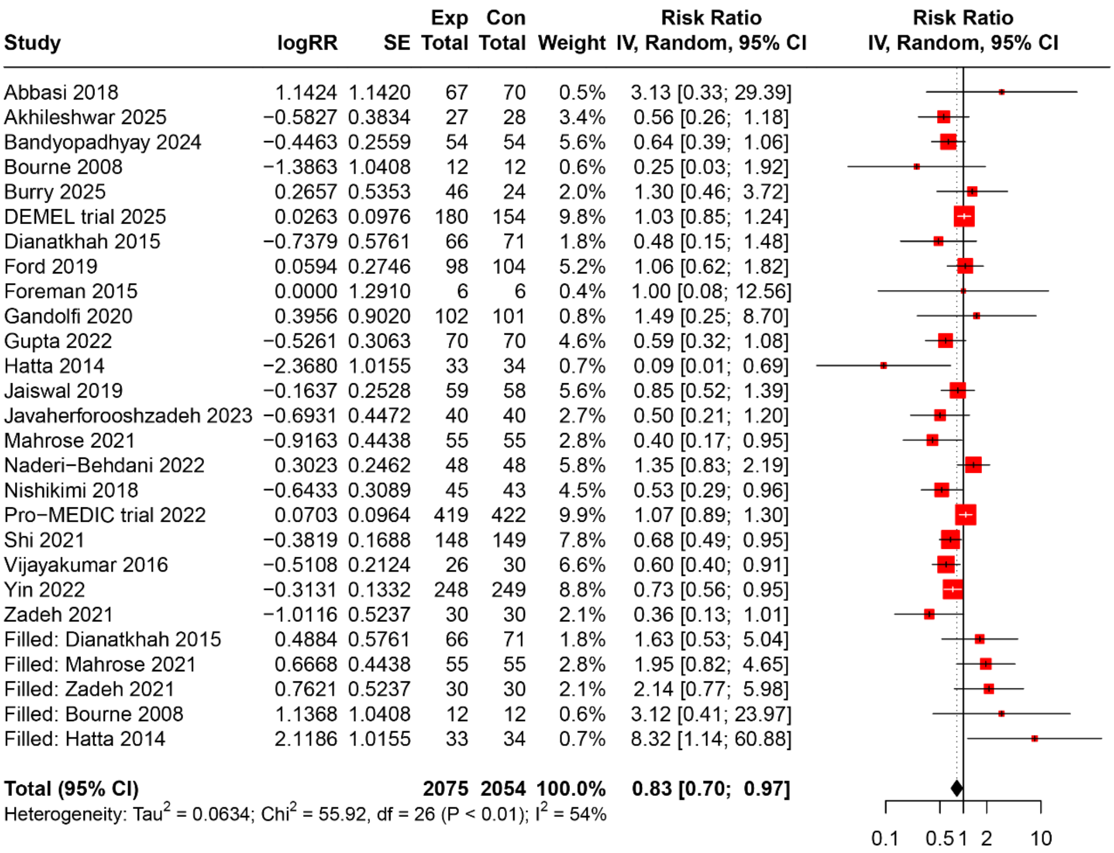

Figure S2: Forest plot of the meta-analysis after trim-and-fill adjustment for delirium

Study	MD	SE	Exp Total	Cont Total	Weight	Mean Difference IV, Random, 95% CI
Abbasi 2018	−1.0000	12.6899	67	70	0.1%	−1.00 [−25.87; 23.87]
Akhileshwar 2025	−1.4500	0.4775	27	28	7.9%	−1.45 [−2.39; −0.51]
Bandyopadhyay 2024	−2.0800	0.8707	54	54	6.4%	−2.08 [−3.79; −0.37]
DEMEL trial 2025	−1.6000	1.2643	147	154	4.9%	−1.60 [−4.08; 0.88]
Gandolfi 2020	0.0000	0.8451	102	101	6.5%	0.00 [−1.66; 1.66]
Jaiswal 2019	0.3300	0.3577	59	58	8.3%	0.33 [−0.37; 1.03]
Javaherforooshzadeh 2023	−0.4700	0.4390	40	40	8.0%	−0.47 [−1.33; 0.39]
Mahrose 2021	−0.5000	0.3292	55	55	8.4%	−0.50 [−1.15; 0.15]
Naderi−Behdani 2022	1.0000	1.6524	48	48	3.7%	1.00 [−2.24; 4.24]
Nishikimi 2018	−3.0800	1.4258	45	43	4.4%	−3.08 [−5.87; −0.29]
Pro−MEDIC trial 2022	0.6700	0.2052	419	422	8.6%	0.67 [0.27; 1.07]
Vijayakumar 2016	−1.7100	1.3554	26	30	4.6%	−1.71 [−4.37; 0.95]
Filled: Akhileshwar 2025	1.9442	0.4775	27	28	7.9%	1.94 [1.01; 2.88]
Filled: DEMEL trial 2025	2.0942	1.2643	147	154	4.9%	2.09 [−0.38; 4.57]
Filled: Vijayakumar 2016	2.2042	1.3554	26	30	4.6%	2.20 [−0.45; 4.86]
Filled: Bandyopadhyay 2024	2.5742	0.8707	54	54	6.4%	2.57 [0.87; 4.28]
Filled: Nishikimi 2018	3.5742	1.4258	45	43	4.4%	3.57 [0.78; 6.37]
**Total (95% CI)**			**1388**	**1412**	**100.0%**	**0.18 [−0.64; 1.00]**

Heterogeneity: $Tau^2 = 1.9965$; $Chi^2 = 71.30$, df = 16 ($P < 0.01$); $I^2 = 78\%$

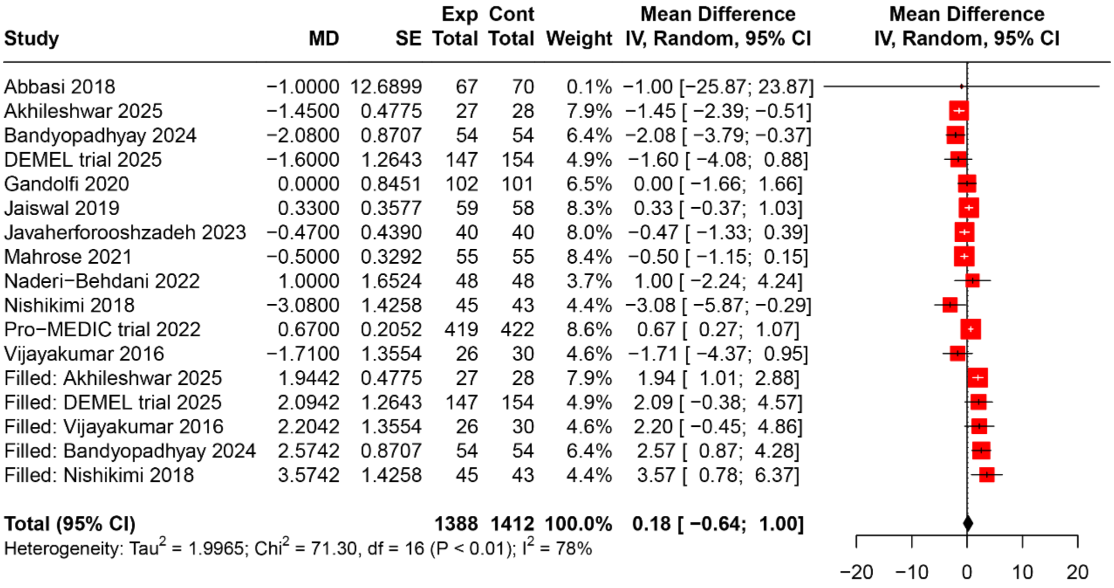

Figure S3: Forest plot of the meta-analysis after trim-and-fill adjustment for length of ICU stay

Study	P−value	Tau2	Tau	I2	Risk Ratio MH, Random, 95% CI
Omitting Abbasi 2018	< 0.01	0.0550	0.2346	51%	0.76 [0.64; 0.89]
Omitting Akhileshwar 2025	< 0.01	0.0541	0.2325	51%	0.77 [0.65; 0.91]
Omitting Bandyopadhyay 2024	< 0.01	0.0573	0.2394	51%	0.77 [0.65; 0.91]
Omitting Bourne 2008	< 0.01	0.0526	0.2294	51%	0.77 [0.65; 0.91]
Omitting Burry 2025	< 0.01	0.0565	0.2377	52%	0.75 [0.63; 0.89]
Omitting DEMEL trial 2025	< 0.01	0.0514	0.2268	47%	0.74 [0.62; 0.87]
Omitting Dianatkhah 2015	< 0.01	0.0533	0.2310	51%	0.77 [0.65; 0.91]
Omitting Ford 2019	< 0.01	0.0583	0.2414	52%	0.74 [0.63; 0.89]
Omitting Foreman 2015	< 0.01	0.0546	0.2336	52%	0.76 [0.64; 0.90]
Omitting Gandolfi 2020	< 0.01	0.0554	0.2354	52%	0.76 [0.64; 0.89]
Omitting Gupta 2022	< 0.01	0.0548	0.2340	51%	0.77 [0.65; 0.91]
Omitting Hatta 2014	< 0.01	0.0495	0.2226	46%	0.78 [0.66; 0.91]
Omitting Jaiswal 2019	< 0.01	0.0620	0.2491	52%	0.75 [0.63; 0.90]
Omitting Javaherforooshzadeh 2023	< 0.01	0.0528	0.2297	51%	0.77 [0.65; 0.91]
Omitting Mahrose 2021	< 0.01	0.0486	0.2205	49%	0.78 [0.66; 0.92]
Omitting Naderi−Behdani 2022	< 0.01	0.0472	0.2173	48%	0.74 [0.63; 0.87]
Omitting Nishikimi 2018	< 0.01	0.0507	0.2252	49%	0.78 [0.66; 0.92]
Omitting Pro−MEDIC trial 2022	< 0.01	0.0443	0.2105	43%	0.73 [0.62; 0.87]
Omitting Shi 2021	< 0.01	0.0611	0.2472	50%	0.77 [0.64; 0.92]
Omitting Vijayakumar 2016	< 0.01	0.0536	0.2314	49%	0.78 [0.65; 0.92]
Omitting Yin 2022	< 0.01	0.0651	0.2552	50%	0.76 [0.63; 0.91]
Omitting Zadeh 2021	< 0.01	0.0496	0.2226	49%	0.78 [0.66; 0.92]
**Total (95% CI)**	**< 0.01**	**0.0539**	**0.2321**	**50%**	**0.76 [0.65; 0.90]**

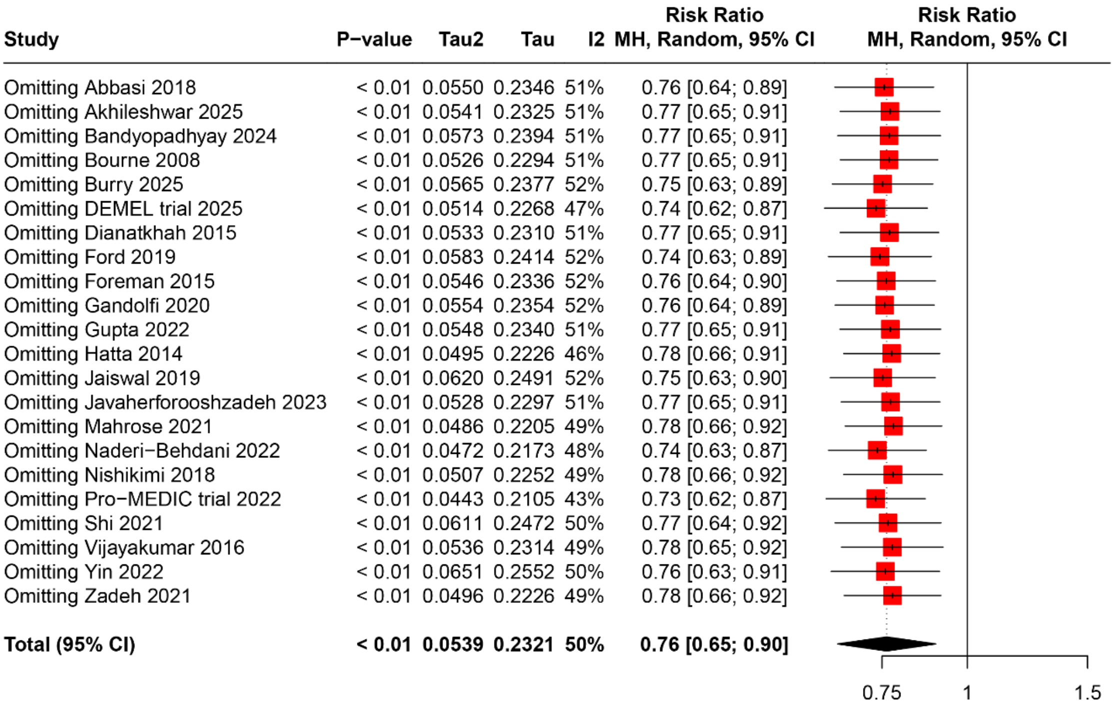

Figure S4: Sensitivity analysis for delirium

Study	P−value	Tau2	Tau	I2	Mean Difference IV, Random, 95% CI
Omitting Abbasi 2018	0.09	0.6593	0.8120	74%	−0.56 [−1.20; 0.09]
Omitting Akhileshwar 2025	0.22	0.5687	0.7541	65%	−0.41 [−1.07; 0.24]
Omitting Bandyopadhyay 2024	0.20	0.5363	0.7323	69%	−0.41 [−1.03; 0.22]
Omitting DEMEL trial 2025	0.13	0.6663	0.8162	73%	−0.51 [−1.17; 0.15]
Omitting Gandolfi 2020	0.08	0.7625	0.8732	74%	−0.63 [−1.34; 0.08]
Omitting Jaiswal 2019	0.05	0.6938	0.8329	73%	−0.71 [−1.41; −0.00]
Omitting Javaherforooshzadeh 2023	0.11	0.8278	0.9098	73%	−0.61 [−1.35; 0.14]
Omitting Mahrose 2021	0.11	0.8417	0.9175	72%	−0.61 [−1.36; 0.15]
Omitting Naderi−Behdani 2022	0.07	0.6989	0.8360	74%	−0.62 [−1.29; 0.05]
Omitting Nishikimi 2018	0.17	0.5605	0.7487	70%	−0.44 [−1.06; 0.18]
Omitting Pro−MEDIC trial 2022	0.02	0.4013	0.6335	46%	−0.73 [−1.34; −0.13]
Omitting Vijayakumar 2016	0.13	0.6609	0.8130	73%	−0.51 [−1.17; 0.15]
**Total (95% CI)**	**0.09**	**0.6586**	**0.8115**	**71%**	**−0.56 [−1.20; 0.08]**

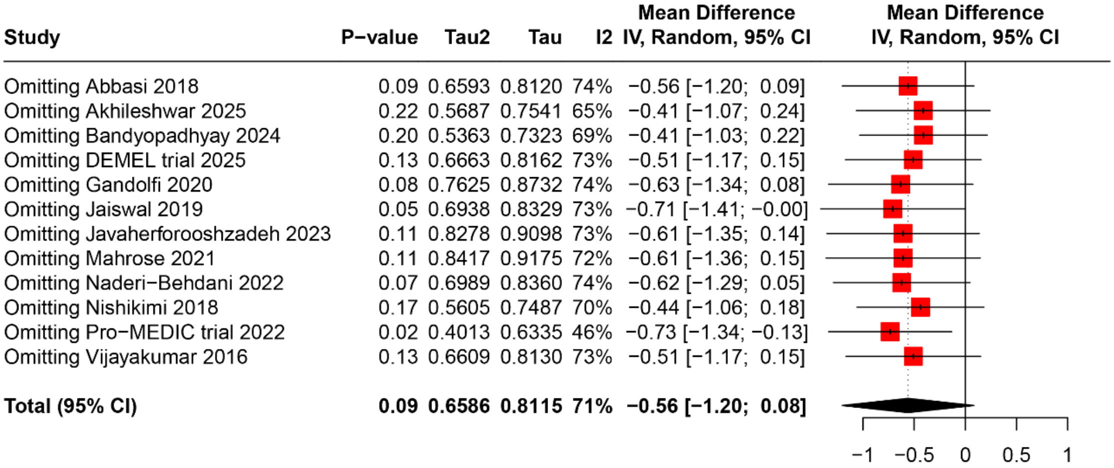

Figure S5: Sensitivity analysis for length of ICU stay

Study	P−value	Tau2	Tau	I2	Mean Difference IV, Random, 95% CI
Omitting Abbasi 2018	0.56	0.2007	0.4480	85%	−0.16 [−0.69; 0.37]
Omitting Akhileshwar 2025	0.29	0	0	0%	0.18 [−0.16; 0.52]
Omitting DEMEL trial 2025	0.40	0.2490	0.4990	80%	−0.29 [−0.95; 0.37]
Omitting Jaiswal 2019	0.22	0.1604	0.4005	55%	−0.36 [−0.93; 0.21]
Omitting Nishikimi 2018	0.68	0.2248	0.4741	86%	−0.12 [−0.70; 0.46]
**Total (95% CI)**	**0.51**	**0.1948**	**0.4414**	**81%**	**−0.17 [−0.69; 0.35]**

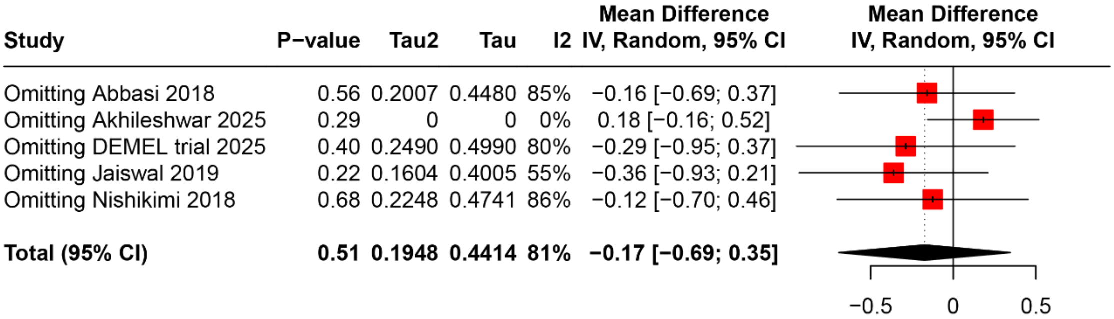

Figure S6: Sensitivity analysis for duration of delirium

Study	P−value	Tau2	Tau	I2	Risk Ratio MH, Fixed, 95% CI
Omitting Abbasi 2018	0.20	0	0	0%	0.91 [0.79; 1.05]
Omitting Bandyopadhyay 2024	0.14	0	0	0%	0.90 [0.78; 1.04]
Omitting Burry 2025	0.33	0	0	0%	0.93 [0.81; 1.07]
Omitting DEMEL trial 2025	0.32	0	0	0%	0.93 [0.79; 1.08]
Omitting Gandolfi 2020	0.29	0	0	0%	0.93 [0.80; 1.07]
Omitting Gupta 2022	0.26	0	0	0%	0.92 [0.80; 1.06]
Omitting Jaiswal 2019	0.27	0	0	0%	0.92 [0.80; 1.06]
Omitting Naderi−Behdani 2022	0.24	0	0	0%	0.92 [0.80; 1.06]
Omitting Nishikimi 2018	0.25	0	0	0%	0.92 [0.80; 1.06]
Omitting Pro−MEDIC trial 2022	0.19	0	0	0%	0.90 [0.77; 1.05]
Omitting Shi 2021	0.29	0	0	0%	0.93 [0.80; 1.07]
Omitting Yin 2022	0.65	0	0	0%	0.96 [0.80; 1.15]
**Total (95% CI)**	**0.25**	**0**	**0**	**0%**	**0.92 [0.80; 1.06]**

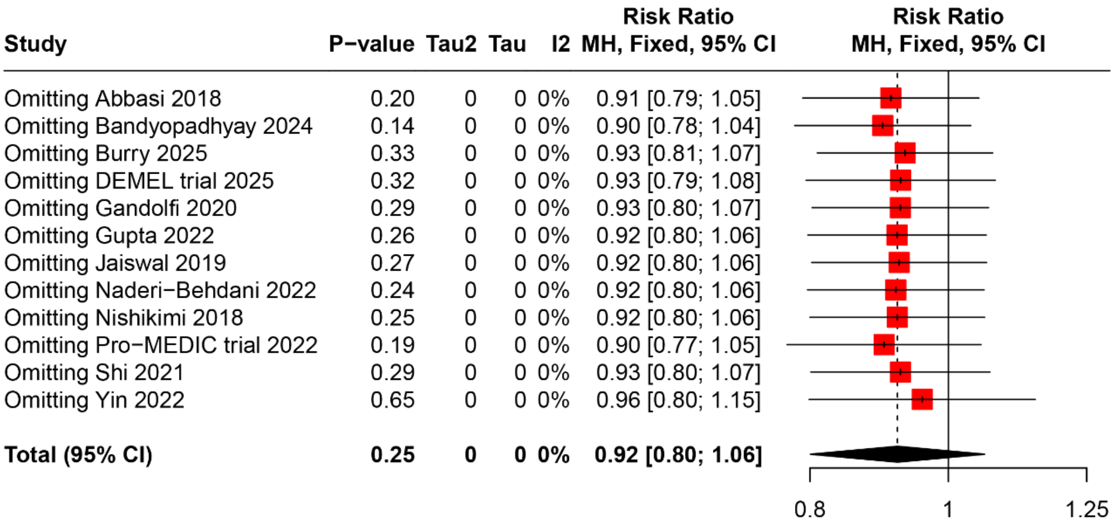

Figure S7: Sensitivity analysis for overall mortality
